# First-line drug-resistant tuberculosis among children under 15 years in Ethiopia: insights from phenotypic and whole-genome sequencing approaches

**DOI:** 10.1186/s12879-026-13206-9

**Published:** 2026-03-31

**Authors:** Yeshiwork Abebaw, Abaysew Ayele, Arash Ghodousi, Dawit Hailu Alemayehu, Gebremedhin Gebremicael, Getu Diriba, Getachew Seid, Andrea Maurizio Cabibbe, Markos Abebe, Anandi Sheth, Rahel Argaw, Woldaregay Erku Abegaz

**Affiliations:** 1 Department of Microbiology, Immunology and Parasitology, College of Health Sciences, Addis Ababa University, Addis Ababa, Ethiopia; 2 Ethiopian Public Health Institute, Addis Ababa, Ethiopia; 3 Armauer Hansen Research Institute, Addis Ababa, Ethiopia; 4 Vita-Salute San Raffaele University, Milan, Italy; 5 IRCCS San Raffaele Scientific Institute, Milan, Italy; 6 Department of Medicine, School of Medicine, Emory University, Atlanta, USA; 7 Department of Pediatrics and Child Health, College of Health Sciences, Addis Ababa University, Addis Ababa, Ethiopia

**Keywords:** Childhood drug-resistant TB, Phenotypic drug susceptibility testing, Whole-genome sequencing

## Abstract

**Background:**

Childhood drug-resistant tuberculosis is often underdiagnosed and inadequately characterized due to the paucibacillary nature of the disease. This study aimed to assess resistance to first-line anti-tuberculosis drugs in children using phenotypic drug susceptibility testing and whole-genome sequencing.

**Methods:**

A retrospective-prospective study was conducted on culture-confirmed childhood tuberculosis cases in Ethiopia (2017–2023). Phenotypic drug susceptibility testing was performed on 110 *Mycobacterium tuberculosis* complex isolates. Whole-genome sequencing was completed for 85 of these isolates, which were analyzed using the TB-Profiler and MTBSeq pipelines. We assessed the sensitivity, specificity, predictive values, and kappa agreement of whole-genome sequencing compared with phenotypic drug susceptibility testing.

**Results:**

Phenotypic resistance to at least one first-line anti-TB drug was observed in 26/110 (23.6%) of the examined isolates, with isoniazid resistance being the most frequent, 23/110 (20.9%), followed by rifampicin resistance, 18/110 (16.4%). TB-Profiler showed almost perfect agreement with phenotypic drug susceptibility testing for rifampicin (sensitivity 94.4%, kappa = 0.96) and isoniazid (sensitivity 91.3%, kappa = 0.91), whereas MTBSeq showed slightly lower performance. Both pipelines demonstrated moderate to weak agreement with phenotypic drug susceptibility testing for detecting resistance to ethambutol, pyrazinamide, and streptomycin. The most frequently observed resistance mutations among phenotypically resistant isolates were *rpoB* (Ser450Leu), *katG* (S315Thr), *embB (*Met306Ile), and *pncA* (C-11 A > G) for rifampicin, isoniazid, ethambutol, and pyrazinamide, respectively. Discrepancies between genotypic and phenotypic drug susceptibility testing were observed across all first-line anti-TB drug-resistant isolates, particularly for ethambutol and pyrazinamide.

**Conclusion:**

We found a high prevalence of isoniazid resistance, along with rifampicin resistance, underscoring the need for early detection in vulnerable groups. Whole-genome sequencing showed good accuracy for these drugs, with TB-Profiler performing best.

**Clinical trial number:**

Not applicable.

**Supplementary Information:**

The online version contains supplementary material available at 10.1186/s12879-026-13206-9.

## Introduction

Drug-resistant tuberculosis (DR-TB) is a major cause of morbidity and mortality among tuberculosis-infected children [1, 2]. A 2025 study showed that global multidrug-resistant tuberculosis (MDR-TB) burden in children and adolescents has increased over the past 30 years [3]. Moreover, a mathematical model study showed that around 850,000 children were estimated to have DR-TB, including 58,000 of them with isoniazid-mono-resistant tuberculosis, 25,000 with MDR-TB, and 1,200 with extensive drug-resistant tuberculosis (XDR-TB) [4]. Childhood DR-TB is also a serious concern in Ethiopia, as demonstrated by recent data that showed 5 out of 77 (6.5%) childhood TB isolates were identified as drug-resistant to at least one of the first-line anti-TB drugs tested [5].

Despite the efforts made globally towards the control of TB, childhood DR-TB is considered a major public health challenge that is often underdiagnosed and poorly characterized due to its paucibacillary nature [6]. Phenotypic drug susceptibility testing (pDST) on solid or liquid media is the gold standard for determining the drug resistance profile of *Mycobacterium tuberculosis* complex (MTBC). However, it needs culture and has a long turnaround time, which may delay treatment initiation [7]. To overcome these challenges, there has been a recent emphasis on improving diagnostic tools and treatment outcomes in children, leading to the development of rapid molecular tests for detecting rifampicin resistance or multidrug-resistant TB (RR/MDR-TB) [7, 8, 9].

Whole-genome sequencing (WGS) has emerged as a powerful tool for a comprehensive approach to identifying genetic mutations associated with resistance to first- and second-line anti-TB drugs and conducting high-resolution outbreak investigations of MTBC [10, 11]. In Ethiopia, WGS has been applied in national and regional studies to characterize the genomic diversity, transmission dynamics, and drug-resistance profiles of MTBC. A nationwide drug resistance survey incorporated WGS to update the prevalence and patterns of drug resistance across multiple regions [12]. Moreover, a recent study on XDR-TB using WGS recommended prioritizing the adoption of genome sequencing in TB control with clear strategies [13]. However, given the high cost and the need for skilled personnel to use WGS, routine clinical use of WGS to replace pDST is currently not feasible in Ethiopia.

Nevertheless, it can be useful for resistance surveillance and research purposes, as it can provide valuable data that may be missed in pDST. For instance, recently, numerous studies have examined the sensitivity and specificity of WGS and identified resistance mutations not captured by conventional diagnostics (pDST) [14]. However, there is limited evidence on the concordance between WGS-predicted resistance profiles and pDST results in Ethiopia, especially in childhood TB cases. Therefore, this study aimed to assess resistance to first-line anti-TB drugs in children using phenotypic drug susceptibility testing and whole-genome sequencing.

## Methods

### Study setting and design

A retrospective–prospective study design was employed from January 2017 to June 2023. The study focused on children aged less than 15 years who were diagnosed with confirmed pulmonary TB in selected health facilities across Ethiopia. This nationwide study included 123 children with culture-confirmed pulmonary TB, comprising 88 cases from the third round of the Ethiopian TB drug resistance survey conducted between 2017 and 2018 [12] and 35 additional culture-confirmed TB cases from 12 selected MDR-TB treatment initiative centers for the study, whose data were collected through 2023. Phenotypic drug susceptibility testing was performed on 110 MTBC isolates from these cases, as the remaining 13 isolates were excluded due to contamination or failure to resuscitate them from frozen storage. However, WGS was attempted for 96 of 110 isolates, and sufficient DNA quality was obtained from all. Of the latter, 85 were successfully sequenced.

## Laboratory analysis

### Culture and phenotypic drug susceptibility test

Sputum or gastric aspirate was processed for culture by digesting and decontaminating using an in-house prepared N-acetyl-L-cysteine–sodium hydroxide (NALC–NaOH) method, and then inoculated onto both Löwenstein–Jensen (LJ) medium and Mycobacteria Growth Indicator Tube (MGIT) (Becton, Dickinson, Sparks, Maryland, USA) following the manufacturer’s protocol [15]. Then pDST for first-line anti-TB drugs was performed on MTBC cultures by using liquid media MGIT and the Mycobacterium Growth Indicator Tube (Bactec, Inc) 960 system, according to the World Health Organization (WHO)-recommended critical concentrations using the following anti-TB drugs: isoniazid (INH) at 0.1 µg/mL; rifampicin (RIF) at 0.5 µg/mL; ethambutol (EMB) at 5.0 µg/mL; pyrazinamide (PZA) at 100 µg/mL; streptomycin (STR) at 1.0 µg/mL [16–17]. Phenotypic drug susceptibility testing was also performed on frozen stored samples after they were returned to room temperature, if no documented drug resistance profile was available. Moreover, any discordant results between phenotypic and genotypic were re-evaluated using repeat pDST to confirm discrepancies.

### DNA extraction and whole-genome sequencing

The DNA was extracted from heat-inactivated MGIT isolates as described in a previously published protocol using the Cetyltrimethylammonium Bromide-lysozyme (CTAB) method [18]. In brief, lysozymes, EDTA, and proteinase K were added to lyse the cell wall, chelate heavy metals, and digest proteins. DNA was subsequently purified using phenol–chloroform extraction and precipitated with isopropanol. The DNA pellet was washed and rehydrated in Tris–EDTA buffer. Quality control of the extracted genomic DNA was performed using a Qubit 2.0 fluorometer (Thermo Fisher Scientific, Waltham, MA, USA), agarose gel electrophoresis, and a NanoDrop (Thermo Fisher Scientific, Waltham, MA, USA) to determine DNA concentration, integrity, size, and quality. High-quality DNA was used for library preparation with the Nextera XT kit (Illumina), and then the Illumina NextSeq 500/550 platform was used for sequencing.

### Bioinformatics analysis

Paired-end raw sequencing reads were quality-checked using FastQC (v0.12.1). Low-quality bases, adapter contamination, and sequences at the start and end of reads were removed through trimming and filtering using Trimmomatic (v0.33) [19]. Duplicate reads were identified and marked using Picard MarkDuplicates v2.18.29 to minimize misalignment artifacts. High-quality paired-end reads, where the Phred quality score was > 20, and the minimum read length was 50 base pairs, were considered for further analysis. Species identification and contamination screening were performed using k-mer-based taxonomic classification with Kraken 2 [20]. The reads were aligned against the *Mycobacterium tuberculosis* H37Rv reference genome (GenBank accession: NC_000962.3) using BWA-MEM (v0.7.16) [21], and mapping quality was evaluated with Samtools stats (v1.19.2) to ensure that the average depth of coverage was greater than 15× and genome coverage exceeded 99% [22]. The TB-Profiler command-line (v6.2.0) and MTBSeq (v1.1.0) pipelines identified genetic markers of antimicrobial resistance. The nucleotide changes detected by MTBSeq were identified as genomic positions. To facilitate comparison with published studies and established resistance databases, these positions were converted into TB-Profiler style annotations using a gene-internal coordinate framework [23]. The identified mutations were then compared to mutations found in the WHO 2023 database, where the mutations are categorized into five WHO catalog groups, depending on the WHO-assigned confidence grade: associated with resistance (Group 1), associated with resistance-interim (Group 2), uncertain significance (Group 3), not associated with resistance-interim (Group 4), and not associated with resistance (Group 5) [24].

### Statistical analyses

Statistical analysis was performed using SPSS v.26.0 (SPSS Inc., Chicago, IL, USA). Descriptive statistics were used to assess the frequency of drug-resistant TB. The sensitivity, specificity, positive predictive value, and negative predictive value of WGS were calculated using the ratio method, with pDST serving as the gold standard. Additionally, Cohen’s kappa statistic was employed to assess the reliability of WGS. The interpretation of the kappa values was as follows: 0-0.2 indicated no agreement, 0.21–0.39 indicated minimal agreement, 0.40–0.59 indicated weak agreement, 0.60–0.79 indicated moderate agreement, 0.80–0.90 indicated strong agreement, and values above 0.90 indicated almost perfect agreement [25]. Statistical significance was determined using the chi-square test or Fisher’s exact test, with *p* < 0.05 considered significant.

## Results

A total of 123 children with culture-positive MTBC isolates were enrolled in the study. Of these, 110 cases had available first-line pDST results. Among the 110 cases, 101 were newly diagnosed patients with no prior history of TB treatment, while the remaining nine cases had a history of previous TB treatment. Phenotypic resistance to at least one first-line anti-TB drug was observed in 26 of 110 isolates (23.6%). Among new cases, 21/101 (20.8%) exhibited resistance, while the remaining 5/9 (55.6%) were among previously treated cases. MDR/RR-TB was detected in 18/110 (16.4%), including 14/101 (13.9%) among new and 4/9 (44.4%) among previously treated cases. Isoniazid resistance was the most common resistance (20.9%) in both new and previously treated cases compared to other first-line drugs, where a statistically significant association was observed with treatment history (*p* = 0.008) (Table 1).

**Table 1:** Phenotypic drug resistance profile of childhood TB isolates among new and previously treated cases

Drug Resistance Profile of Isolates		Total (%) *n* = 110,	History of treatment		p-value
			New (%) *n* = 101	Previously treated (%) *n* = 9	
Drug resistance to at least one first-line anti-TB drug	Resistance	26 (23.6)	21 (20.8)	5 (55.6)	0.019
	Susceptible	84 (76.4)	80 (79.2)	4 (44.4)	
MDR-TB/RIF	Resistance	18 (16.4)	14 (13.9)	4 (44.4)	0.017
	Susceptible	92 (83.6)	87 (86.1)	5 (55.6)	
INH	Resistance	23 (20.9)	18 (17.8)	5 (55.6)	0.008
	Susceptible	87 (79.1)	83 (82.2)	4 (44.4)	
EMB	Resistance	13 (11.8)	11 (10.9)	2 (22.2)	0.313
	Susceptible	97 (88.2)	90 (89.1)	7 (77.8)	
STR	Resistance	15 (13.6)	11 (10.9)	4 (44.4)	0.019
	Susceptible	95 (86.4)	90 (89.1)	5 (55.6)	
PZA	Resistance	11 (10.0)	9 (8.9)	2 (22.2)	0.402
	Susceptible	96 (87.3)	89(88.1)	7(77.8)	
	Not done	3(2.7)	3(3.0)	0	

Abbreviations: n = Number of participants; INH = Isoniazid; RIF = Rifampicin; EMB = Ethambutol; STR = Streptomycin; PZA = Pyrazinamide

### Performance characteristics of WGS in comparison with phenotypic DST

By considering pDST as the gold standard, we evaluated the performance of WGS using the TB-Profiler and MTBSeq pipelines to predict resistance to first-line anti-TB drugs (Table 2). The analysis showed that TB-Profiler demonstrated 94.4% sensitivity and 100% specificity for detecting RIF resistance, and 91.3% sensitivity and 98.4% specificity for detecting INH resistance. The kappa values indicated almost perfect agreement between pDST and TB-Profiler for both RIF and INH resistance (0.96 and 0.91, respectively). On the other hand, the MTBSeq pipeline demonstrated slightly lower performance, with 94.4% sensitivity and 97.0% specificity for RIF resistance, and 82.6% sensitivity and 98.4% specificity for INH resistance. Even then, the MTBSeq pipeline’s agreement with pDST remained strong, with kappa values of 0.89 for RIF and 0.84 for INH, although the TB-Profiler outperforms for both of these drugs. However, for EMB, PZA, and STR resistance detection, both pipelines showed lower to moderate agreement compared with their strong performance for RIF and INH, as shown in Table 2. Furthermore, a significant reduction in the prediction of PZA resistance was noted from both pipelines. However, the MTBSeq pipeline had lower sensitivity and a kappa value of 0.39 compared to TB-Profiler (0.62), indicating weak agreement between the pipelines and pDST for this particular drug.

**Table 2:** Performance characteristics of WGS of drug resistance detected by TB-Profiler and MTBSeq compared to pDST

Drug	TB-Profiler					MTBSeq				
	Sensitivity in %	Specificity in %	PPV in %	NPV in %	Kappa (*p*-value)	Sensitivity in %	Specificity in %	PPV in %	NPV in %	Kappa (*p*-value)
RIF	94.4	100	100	98.5	0.96 (< 0.001)	94.4	97.0	88.9	98.5	0.89(< 0.001)
INH	91.3	98.4	95.5	96.8	0.91 (< 0.001)	82.6	98.4	95.0	93.8	0.84(< 0.001)
EMB	84.6	95.8	78.6	97.2	0.78 (< 0.001)	83.3	95.8	76.9	97.2	0.76(< 0.001)
PZA	63.6	95.9	70.0	94.7	0.62 (< 0.001)	30.0	98.6	75.0	91.4	0.39(< 0.001)
STM	86.7	94.3	76.5	97.1	0.77 (< 0.001)	64.3	95.7	75.0	93.3	0.64(< 0.001)

Abbreviation: INH = Isoniazid; RIF = Rifampicin; EMB = Ethambutol; STR = Streptomycin; PZA = Pyrazinamide pDST: phenotypic Drug Susceptible Test, WGS: Whole-genome sequencing, PPV: Positive Predictive Value, NPV: Negative Predictive Value

### Drug resistance-associated mutations detected by TB-Profiler and MTBSeq among phenotypically resistant isolates for first-line drugs

**Fig. 1:**
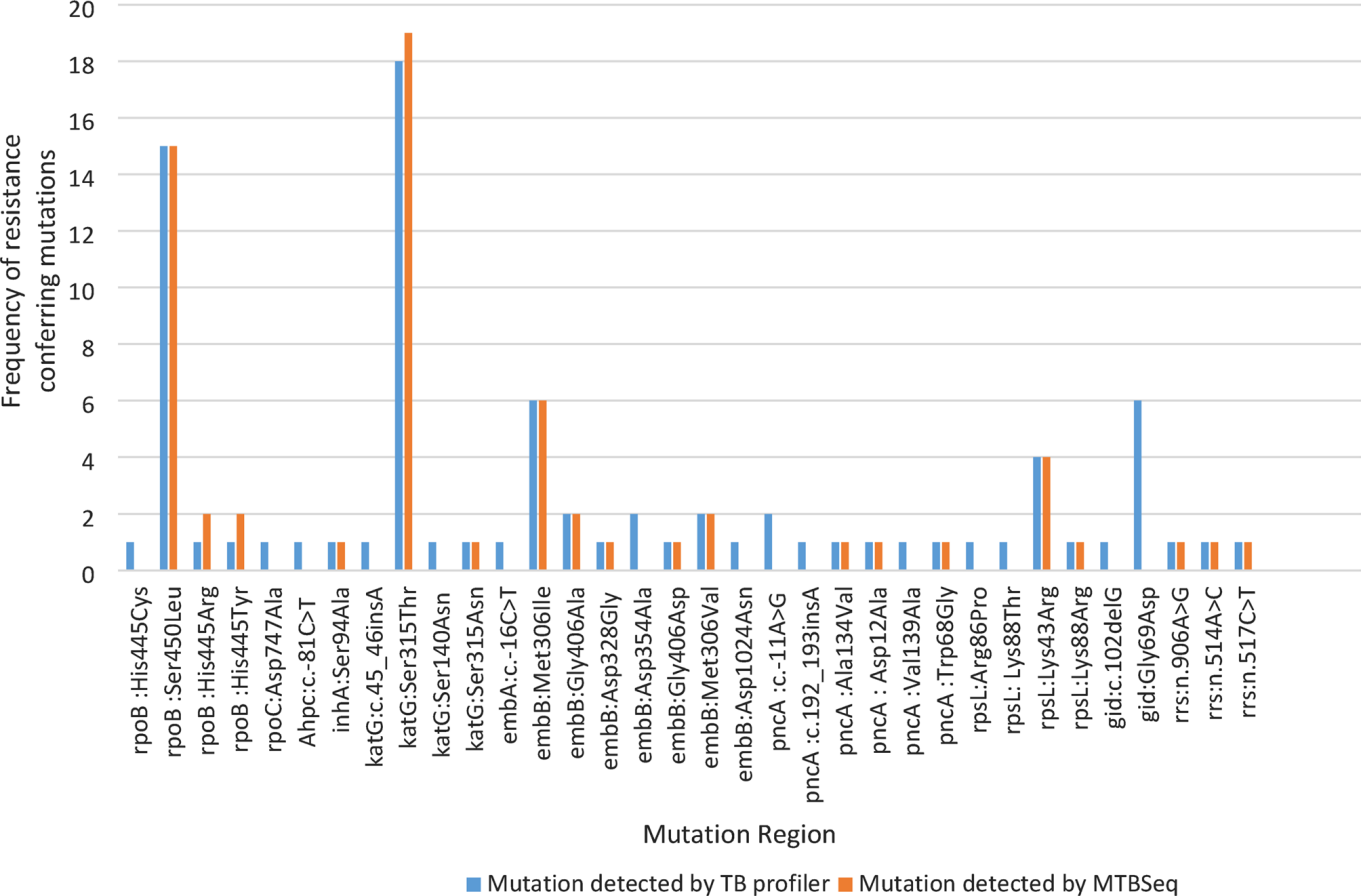
Comparison of the frequency of drug resistance-associated mutations detected by TB-Profiler and MTBSeq among phenotypically resistant TB isolates from children

This study compared the frequency of drug resistance mutations detected by TB-Profiler and MTBSeq (Fig. 1). The most frequent RIF resistance-conferring mutation, detected by both TB-Profiler and MTBSeq, was at the *rpoB* gene (Ser450Leu) in 15/17 (88.2%) of the resistant samples. Notably, 10 of these mutations occurred in Lineage 4.2.2. However, some RIF-associated gene mutations, such as His445Cys, and compensatory mutations, such as p.Asp747Ala at *rpoC*, were missed by MTBSeq but detected by TB-Profiler. Similarly, some INH-associated mutations, such as c.45_46insA and p.Ser140Asn, were identified by TB-Profiler but were not detected by MTBSeq, and they were not present in the WHO 2023 catalog. Moreover, high INH-resistance-conferring mutations were at *katG (*Ser315Thr) in 18 of the 21 (85.7%) resistant isolates, with 12 among lineage 4.2.2 and 5 among lineage 3, as determined by TB-Profiler. In addition, 18/19 (94.7%) INH-resistant strains harbor the *katG* (Ser315Thr) mutation, detected by MTBSeq, among five isolates of lineage 3, 11 from lineage 4.2.2, and one each from lineages 4.2.1 and 4.1.2. (Supplement Tables 1 and 2). The most frequent EMB-associated resistance mutation detected by both pipelines was Met306Ile at the *embB* gene: 6/11 (54.5%) by TB-Profiler and 6/10 (60.0%) by MTBSeq. On the other hand, while seven of the PZA-associated resistance mutations, mainly at *pncA*, were detected by TB-Profiler, only three mutations were identified by the MTBSeq pipeline (Fig. 1). Likewise, some STR-associated mutations, such as *gid* (Gly69Asp), were detected by TB-Profiler only (Fig. 1).

### Drug resistance-conferring mutations detected by TB-Profiler among phenotypically susceptible isolates

TB-Profiler detected drug resistance-conferring mutations in all first-line drugs, except RIF, in some phenotypically susceptible isolates. In this connection, one isolate for INH, three for EMB and PZA, and four for STR were identified genotypically as resistant (Table 3). An INH resistance-conferring mutation was observed in lineage 4.2.1 within the *katG* gene (c.597dupC), which is not recognized in the WHO catalog. A discrepancy between the TB-Profiler and the WHO catalog also occurred for the EMB resistance mutation at the *embB* gene, which primarily occurred in lineage 3, where three MDR-TB isolates each exhibited three mutations (Gly406Ala, p.Met306Ile, and p.Met306Val). Whereas TB-Profiler identified these mutations as missense variants, the WHO’s catalog classified them as Group 1 EMB mutations conferring resistance (Table 3). In the *pncA* gene, which is associated with PZA resistance, TB-Profiler identified a missense mutation at Val139Ala in a lineage 3 MDR-TB isolate, and a nonsense mutation (Tyr41*) in two MDR-TB isolates from lineage 4.2.2. Val139Ala was classified in the WHO catalog as a Group 1 EMB resistance-conferring mutation, and the Tyr41* mutation was identified as “Not classified” by the catalog. Regarding STR resistance-conferring genes, two MDR-TB isolates belonging to lineage 4.2.2 harbored a *gid* mutation (Gly69Asp), which the WHO classified as of uncertain significance. Another heteroresistant mutation was observed in one STR mono-resistant lineage 3 isolate, in the *rrs* gene at positions 799 C > T, which is not classified in any group in the WHO’s catalog (Table 3).

**Table 3:** Drug-resistant-associated mutations detected by TB-Profiler in phenotypically susceptible isolates, compared to the WHO catalogue

First-line drug	Genotypic resistance	Gene	Mutation detected by TB-Profiler				Confidence Grading by WHO
			Amino acid change	Allele freq(%)	Variant	Lineage	
INH	1	*katG*	597dupC	100	Frameshift	Lineage 4.2.1	Not classified
EMB	1	*embB*	Gly406Ala	100	Missense	Lineage 3	Group 1
	1	*embB*	Met306Ile	100	Missense	Lineage 3	Group 1
	1	*embB*	Met306Val	100	Missense	Lineage 3	Group 1
PZA	2	*pncA*	Tyr41*	100	Stop Gained	Lineage 4.2.2	Not classified
	1	*pncA*	Val139Ala	100	Missense	Lineage 3	Group 1
STM	1	*rrs*	n.799 C > T	27.2	Non_Coding Region	Lineage 3	Not classified
	1	*rrs*	n.514 A > T	100	Non-Coding Transcript Exon	Lineage 4.2.2	Not classified
	2	*Gid*	Gly69Asp	100	Missense	Lineage 4.2.2	Group 3

Abbreviations: n = Number of participants; INH = Isoniazid; RIF = Rifampicin; EMB = Ethambutol; STR = Streptomycin; PZA = Pyrazinamide

### Mutation detected by TB-Profiler among isolates exhibiting phenotypic resistance without known genotypic markers

Isolates determined to be drug-resistant by phenotypic DST but identified as susceptible by the TB-Profiler were further examined for mutations in the target regions. Among these, some isolates showed mutations in the drug target regions, while others had none detectable (Table 4). For example, in one of the two isolates with phenotypic mono-resistance to INH but genotypic susceptibility by TB-Profiler, two mutations were identified: *katG (*Arg463Leu) and − 88G > A mutation in the promoter region of *ahpC*. However, the WHO catalog classifies both as not associated with INH resistance. In contrast, another isolate exhibited phenotypic resistance to both INH and RIF; however, TB-Profiler detected no resistance-associated mutations in the resistance-associated loci. Multiple missense mutations were observed in one EMB mono-resistant isolate, and one MDR-TB isolate resistant to EMB. However, none of these latter mutations were classified as resistance-associated by both the TB-Profiler and the WHO catalog. Moreover, they were also found in the phenotypically susceptible isolates. Similarly, in one PZA-resistant MDR-TB isolate (Lineage 3), an upstream mutation in *pncA* (-125delC) was detected. This mutation was also present in both PZA-resistant and susceptible isolates of the same lineage and is not listed in the WHO catalog, which may indicate that it is a natural or lineage-specific marker (Table 4).

**Table 4:** Mutations detected in the target region by TB-Profiler among phenotypically resistant but TB-Profiler susceptible, and compared with the WHO catalog

First-line drug	Phenotypic resistance (*n*)	Mutation (*n*)	Gene	Mutation detected by TB-Profiler				Confidence Grading by WHO
				Amino acid change	Allele Frequency(%)	Variant	Lineage	
INH	2	1	*katG*	Arg463Leu	100	Missense	Lineage 3	Group 5
			*ahpc*	88G > A	100	Upstream gene	Lineage 3	
EMB	*2*	1	*embR*	Phe376Leu Cys372Gly	11.2+ 13.3	Missense	Lineage 4.2.2	Group 5
				Phe376Leu+Cys372Arg+ Cys372Gly	17.2+ 18.4+ 18.4	Missense	Lineage 4.1.1.3	Group 5
		1	*embc*	Val981Leu	100	Missense	Lineage 4.1.1.3	
PZA	4	1	*pncA*	-125delC	100	Upstream gene	Lineage 3	Not Identified
STM	*2*	1	*rr*	− 187 C > T	100	Upstream gene	Lineage 4 0.6.2	Group 5
		1	*rpsL*	c.-165T > C	100	Upstream gene	Lineage 4.6.2	Group 5

Abbreviations: n = Number of participants; INH = Isoniazid; RIF = Rifampicin; EMB = Ethambutol; STR = Streptomycin; PZA = Pyrazinamide

## Discussion

Diagnosing tuberculosis and detecting drug resistance in children remains a significant challenge due to inadequate sample collection [26]. In the present study, the prevalence of MDR-TB was 16.4%. This estimate is lower than that reported in a systematic review of MDR-TB among TB co-infected populations (20%) [27] but higher than the prevalence reported in a systematic review by Reta et al. on drug-resistant TB in Ethiopia (10.78%) [28] and the third national drug resistance survey, which reported MDR-TB prevalence of 1.28% among new cases and 8.4% among previously treated cases in the general population [12]. These variations in MDR-TB prevalence may be attributed to differences in study populations, sampling strategies, and geographic settings. In our study, some participants were enrolled from MDR-TB treatment initiation centers, which may have increased the probability of detecting RR/MDR-TB.

Improving the diagnosis, treatment decisions, and surveillance strategies against DR-TB in this vulnerable population is critical. In Ethiopia, management of DR-TB follows national guidelines aligned with WHO’s recommendations. Isoniazid-mono-resistant TB is treated with a 6-month regimen of RIF, ETH, PZA, and Levofloxacin. In contrast, RR/MDR-TB is managed with standardized, shorter (9–11 months) or longer all-oral regimens that include Bedaquiline and fluoroquinolones [29]. Other mono-resistance patterns are treated according to pDST results and national protocols. Treatment is primarily standardized but individualized when clinically indicated [29]. Whole-genome sequencing is not currently used for routine clinical decision-making in the Ethiopian national tuberculosis program. Therefore, treatment regimens were determined based on pDST results in accordance with national TB treatment guidelines. Consequently, any discrepancies between phenotypic and genotypic resistance patterns were identified during the research analysis and did not influence patient management. However, WGS may serve as a complementary tool in the future to help resolve such discrepancies and improve the detection of drug resistance [13, 28].

Recently, highly sensitive and specific molecular tests, such as GeneXpert, have been used as primary diagnostic tools, primarily for detecting MTBC and RIF resistance [9]. Recent studies [30], including ours, have shown a rising prevalence of resistance to first-line drugs, particularly to INH. Our findings showed that INH resistance prevalence was higher than RIF resistance (20.9% vs. 16.4%), and both were associated with treatment history, with a statistically significant difference. INH resistance in children is widespread, yet poorly quantified, and poses a substantial barrier to effective tuberculosis treatment. Therefore, improving access to timely drug susceptibility testing for both INH and RIF, along with improved management of latent TB infection in children, is essential [30, 31].

Several bioinformatics tools have recently been developed for analyzing MTBC sequencing data, including TB-Profiler, MTBSeq, KvarQ, TGS-TB, Mykrobe, PhyResSe, and SAM-TB [32, 33]. The current study evaluated the sensitivity and specificity of WGS-based drug susceptibility testing using TB-Profiler and MTBSeq for first-line anti-TB drugs. TB-Profiler showed almost perfect agreement with pDST, with 94.4% sensitivity and 100% specificity for RIF, and 91.3% sensitivity and 96.8% specificity for INH. These results are consistent with previously reported WGS-based molecular DST performance, which showed sensitivities ranging from 91.3% to 97.5% and specificities ranging from 93.6% to 99.0% [34, 35]. MTBSeq results for RIF and INH also aligned with findings from Israel, though moderate differences were noted. Our study reported lower INH sensitivity than that from Israel (82.5% vs. 100%) [33], while it was more in line with data from Eastern China [36]. The sensitivity of MTBSeq for detecting RIF resistance in our study (94.4%) was slightly higher than that reported in the Israel study (75%) [33]. Sensitivity for PZA, EMB, and STR-resistance detection was similar between TB-Profiler and MTBSeq and was moderate to weak, as reported previously. Moreover, both platforms exhibited high specificity for the drugs mentioned above, as observed in previous studies [31, 33, 34].

We also compared the resistance-conferring mutations detected by TB-Profiler and MTBSeq among phenotypically proven isolates resistant to first-line anti-TB drugs. TB-Profiler identified additional mutations compared to MTBSeq, such as resistance genes for RIF at *rpoC*, INH at *katG* and *AhpC*, and PZA at *pncA*. Other authors have also reported that TB-Profiler performed well across most anti-TB drugs [37], while MTBSeq is known to provide a more comprehensive resistance profile [38]. Regardless of the pipeline used, this study observes that most of the mutations for RIF were at the *rpoB* gene (Ser450Leu), for INH at *katG* (Ser315Thr), for EMB at the *embB* gene (Met306Ile), and for PZA at the *pncA* gene, which highlights the importance of these genomic regions for the prediction of drug resistance [39].

Another interesting observation was that an isolate was phenotypically susceptible but carried a less well-known mutation (597dupC), a duplication of a cytosine nucleotide at position 597 of the resistance hotspot gene *katG*. Such duplications can cause a frameshift mutation and lead to a loss or reduction in the catalase-peroxidase enzymatic activity required to activate INH. The lack of association between the detection of this mutation and the absence of phenotypic resistance by pDST could be due to the possibility of low- to moderate-level resistance induced by this mutation under the tested critical concentration, which the WHO recommends for INH (0.1 µg/ml) [16].

Genotypic resistance to EMB and PZA, but phenotypic susceptibility, was observed in our study. Resistance-conferring mutations for EMB (Gly406Ala, Met306Ile, and Met306Val) were identified at the *embB* gene. A previous study shows that mutations in embB406 and 306 with low-level EMB resistance are undetectable at the recommended critical concentration (5 µg/mL) [40]. These findings suggest that pDST may be less reliable for detecting EMB resistance [39], highlighting the WGS method’s greater effectiveness in detecting drug resistance genes than pDST at that critical concentration [41]. On the other hand, examining the drug resistance profile by lineage may be more informative, as all EMB discrepancies observed in this study were found only in lineage 3, even though only three isolates exhibited this profile.

Another important discrepancy between pDST and genotypic resistance detection is that some isolates exhibited phenotypic resistance to first-line anti-TB drugs. Yet, no known resistance-conferring mutations were detected in any of the relevant genes. However, in most of these phenotypically resistant but genotypically susceptible isolates, mutations that were not previously identified as resistance-conferring were detected in genes already recognized as hotspots for resistance. Examples of these mutations include *ahp c* (88G > A) at *katG (Arg463Leu)* for INH; p.Phe376Leu, p.Cys372Gly, p.Phe376Leu, p Cys372Arg, and p. Cys372Gly at *embR* for EMB; (-125delC) at *pncA* for PZA; (-187 C > T) at *rrs*, and (c.-165T > C) at *rpsL* for STR. These mutations were either classified as non-resistance-conferring, of uncertain significance, or not identified at all in the WHO catalog [24]. Studies showed that these types of mutations may enhance survival under drug pressure, for example, by way of altering expression of detoxification pathways as observed in a study which documented isolates exhibiting phenotypic resistance but genotypic susceptibility to INH (*ahpC*,* katG* regulators); these isolates had a mutation at *katG* (Arg463Leu) and *ahpc* (88G > A) [42]. Some isolates in the current study also had no detectable mutations at all, even though they were phenotypically resistant (as observed in one INH, one EMB, and three PZA drug-resistant isolates), suggesting the possible presence of unknown resistance mechanisms or heteroresistance and/or mutations in other drug-resistance-associated loci that indirectly affect susceptibility [43]. Besides, phenotypic DST for EMB and PZA is known to yield inconsistent results [14].

The limitation of this study is that most of the isolates used were from stored samples. The yield of viable isolates recovered from these stored samples was lower than expected. However, to our knowledge, this is the first study of children in Ethiopia to provide baseline information on DR-TB using both WGS and pDST simultaneously.

## Conclusion

We found a high prevalence of INH resistance, along with RIF resistance, underscoring the need for early detection in vulnerable groups. WGS showed good accuracy for these drugs, with TB-Profiler performing best. Discrepancies between genotypic and phenotypic drug susceptibility testing were observed across all first-line anti-TB drug-resistant isolates, mainly for ethambutol and pyrazinamide, which may be due to mutations conferring low-level resistance, mutations of uncertain significance, lineage-specific mutations, and new resistance loci, underscoring the complexity of resistance and the need for further research. Integrating WGS with pDST could enhance the surveillance and detection of drug-resistant TB, thereby improving the management of childhood DR-TB in Ethiopia. We also recommend conducting validation studies on the feasibility of direct WGS detection from sputum or by other targeted sequencing methods, such as Deeplex and Nanopore-based methods, to bypass culture, which remains a major bottleneck in childhood TB diagnosis.

## Supplementary Information

Below is the link to the electronic supplementary material.

Supplementary Material 1

## Data Availability

The datasets generated and analyzed during this study are available from the corresponding author upon reasonable request. All relevant data supporting the findings are included in the manuscript and its supplementary files. The raw sequencing data have been submitted to NCBI as FASTQ files under Accession Numbers: PRJNA1104194 and PRJNA1204469.

## Abbreviations

DR-TB: Drug-resistant tuberculosis
EETB-RTP: Ethiopia-Emory TB Research Training Program
EMB: Ethambutol
INH: Isoniazid
MDR-TB: Multidrug-resistant tuberculosis
MGIT: Mycobacteria Growth Indicator Tube
RIF: Rifampicin
pDST: Phenotypic drug susceptibility testing
PZA: Pyrazinamide
STR: Streptomycin
WHO: World Health Organization
WGS: Whole-genome sequencing

## References

[1] World Health Organization. Global tuberculosis executive summary Report. WHO/CDS/TB/2018.25.

[2] Ramírez-Rueda RY. *Mycobacterium tuberculosis*: clinical and microbiological aspects. The microbiology of respiratory system infections. Elsevier Inc.; 2016. pp. 153–66.

[3] Zhong Y, Xie H, Cai F, et al. Global burden of multidrug-resistant tuberculosis in children and adolescents. Pediatr Res. 2025;98:901–8. 39934644 10.1038/s41390-025-03917-1 PMC12507668

[4] Dodd PJ, Sismanidis C, Seddon JA. Global burden of drug-resistant tuberculosis in children: a mathematical modelling study. Lancet Infect Dis. 2016;3099(16):1–9. 10.1016/S1473-3099(16)30132-3 27342768

[5] Abebaw Y, Abebe M, Tola HH, et al. Pulmonary tuberculosis case notification and burden of drug resistance among children under 15 years of age in Ethiopia: sub-analysis from the third-round drug resistance tuberculosis survey. BMC Pediatr. 2023;23(1):1–10. 37620787 10.1186/s12887-023-04240-6 PMC10463301

[6] Zhuang Z, Sun L, Song X, et al. Trends and challenges of multi-drug resistance in childhood tuberculosis. Front Cell Infect Microbiol. 13:1183590. 10.3389/fcimb.2023.1183590 PMC10275406 37333849

[7] Palomino JC, Martin A, Portaels F, et al. Evaluation of the Automated BACTEC MGIT 960 System for Testing Susceptibility of. J Clin Microbiol. 2007;37(3):607–10.

[8] World Health Organization. Rapid Implementation of the Xpert MTB / RIF diagnostic test: Technical and Operational’ How-to’.

[9] Dunn JJ, Starke JR, Revell PA. Laboratory diagnosis of *Mycobacterium tuberculosis* infection and disease in children. J Clin Microbiol. 2016;54:1434–41. 26984977 10.1128/JCM.03043-15 PMC4879301

[10] Sun W, Gui X, Wu Z, Zhang Y, Yan L. Prediction of drug resistance profile of multidrug-resistant *Mycobacterium tuberculosis* (MDR - MTB) isolates from newly diagnosed cases by whole genome sequencing (WGS): a study from a high tuberculosis burden country. BMC Infect Dis. 2022;1–10. 10.1186/s12879-022-07482-4 PMC9137048 35624432

[11] Liu D, Huang F, Zhang G, et al. Whole-genome sequencing for surveillance of tuberculosis drug resistance and determination of resistance level in China. Clin Microbiol Infect. 2022;28(5):731.e9-731.e15. 10.1016/j.cmi.2021.09.014 34600118

[12] Moga S, Gtahun M, Mohammed Z, et al. The ethiopian third national tuberculosis drug resistance survey incorporating whole genome sequencing. Open Forum Infect Dis. 2025;12(7):. 10.1093/ofid/ofaf367 PMC12278268 40693102

[13] Diriba G, Mollalign H, Meaza A, et al. Whole genome sequencing-based detection of extensively drug-resistant tuberculosis from Ethiopia. Commun Med. 2026;6(14). 10.1038/s43856-025-01271-1 PMC12780197 41354901

[14] Kurtzhals ML, Norman A, Svensson E et al. Applying whole genome sequencing to predict phenotypic drug resistance in *Mycobacterium tuberculosis*: leveraging 20 years of nationwide data from Denmark. Antimicrob Agents Chemother. 2024;68(8). 10.1128/aac.00430-24 PMC11304686 38904390

[15] Salman S, Rüsch-Gerdes S. MGIT Procedure Manual, Mycobact. Growth Indic. Tube Cult. Drug Susceptibility Demonstr. Proj., no. July, pp. 24–40, 2006. Available: http://www.finddiagnostics.org/export/sites/default/resource-centre/find_documentation/pdfs/mgit_manual_nov_2007.pdf (access date Dec 20,2025).

[16] World Health Organization. Technical report on critical concentrations for TB drug susceptibility testing of medicines used in the treatment of drug-resistant TB. WHO. 2018;1–106.

[17] World Health Organization. Technical report on critical concentrations for drug susceptibility testing of isoniazid and the rifamycins (Rifampicin, rifabutin, and rifapentine). Geneva: World Health Organization. 2021. Licence: CC BY-NC-SA 3.0 IGO.

[18] Mannheim B, Protocol. Isolation of high molecular weight genomic DNA from Mycobacteria (CTAB procedure), aliquots at -20 ° C for no longer than one year. Notes–4.

[19] Bolger AM, Lohse M, Usadel B, Trimmomatic. A flexible trimmer for Illumina sequence data. Bioinformatics. 2014;30(15):2114–20. 24695404 10.1093/bioinformatics/btu170 PMC4103590

[20] Wood D, Lu J, Langmead B. 2020. 2020. Kraken 2 taxonomic sequence classification system. Operating Manual. Available from: https://github.com/DerrickWood/kraken2/wiki/Manual. (accessed on Aug 12, 2025).

[21] Li H, Durbin R. Fast and accurate short read alignment with Burrows-Wheeler transform. Bioinformatics. 2009;25(14):1754–60. 19451168 10.1093/bioinformatics/btp324 PMC2705234

[22] Li H, Handsaker B, Wysoker A, et al. The sequence alignment / map format and SAMtools. Bioinformatics 25(16):2078–9. 10.1093/bioinformatics/btp352 PMC2723002 19505943

[23] Kapopoulou A, Lew JM, Cole ST. The MycoBrowser portal: a comprehensive and manually annotated resource for mycobacterial genomes. Tuberculosis. 2011;91(1):8–13. 20980200 10.1016/j.tube.2010.09.006

[24] World Health Organization. Catalogue of mutations in *Mycobacterium tuberculosis* complex and their association with drug resistance, second edition. Geneva: World Health Organization; *2023. Licence: CC BY-NC-SA 3.0 IGO.*

[25] Abebe G, Bonsa Z, Kebede W. Treatment Outcomes and Associated Factors in Tuberculosis Patients at Jimma University Medical Center: A 5-Year Retrospective Study. Gemeda. Int J Mycobacteriology. 2017;6(3):239–45. 10.4103/ijmy.ijmy_177_18 30860177

[26] Elhassan MM, Elmekki MA, Osman AL, Hamid ME. International Journal of Infectious Diseases Challenges in diagnosing tuberculosis in children: a comparative study from Sudan. Int J Infect Dis. 2016;43:25–9. 26701818 10.1016/j.ijid.2015.12.006

[27] Hailu BK, Demessie Y, Gessese TA, et al. Multi-drug Resistance Tuberculosis in the Context of Co-Infection in Ethiopia: A Systematic Review and Meta-Analysis. JEpidemiol Glob Health. 2025;15(1):19. 39909956 10.1007/s44197-025-00360-7 PMC11799485

[28] Reta AM, Tamene AB, Abate BB et al. *Mycobacterium tuberculosis* Drug Resistance in Ethiopia: An Updated Systematic Review and Meta-Analysis. Trop Med Infect Dis 2022;7(10):300. 36288041 10.3390/tropicalmed7100300 PMC9611116

[29] National guidelines on clinical. and programmatic management of TB, TB/HIV, DR-TB and Leprosy in Ethiopia; 7th edition. FMOH. 2021.

[30] Yuen MC, Tolman WA, Cohen T, et al. Isoniazid-resistant tuberculosis in children: a systematic review. Pediatr Infect Dis J. 2013;32(5):e217-26. 10.1097/INF.0b013e3182865409 PMC3709006 23348808

[31] Worku Y, Getinet T, Mohammed S, et al. Drug-resistant tuberculosis in Ethiopia: Characteristics of cases in a referral hospital and the implications. Int J Mycobacteriol. 2018;7:167–72. 29900895 10.4103/ijmy.ijmy_48_18

[32] Morey-Leon G, Mejı´a-Ponce PM, Granda Pardo JC, Muñoz-Mawyin K, Ferna’ndez-Cadena JC, Garcı´a-Moreira E, et al. A precision overview of genomic resistance screening in Ecuadorian isolates of *Mycobacterium tuberculosis* using web-based bioinformatics tools. PLoS ONE. 2023;18(12):e0294670. 38051742 10.1371/journal.pone.0294670 PMC10697571

[33] Losev Y, Rubinstein M, Nissan I, Haviv P, Barsky Y, Volinsky M, et al. Genomic, phenotypic, and demographic characterization of *Mycobacterium tuberculosis* in Israel in 2021. Front Cell Infect Microbiol. 2023;13:1–11. 10.3389/fcimb.2023.1196904 PMC10622789 37928179

[34] Vīksna A, Sadovska D, Berge I, et al. Genotypic and phenotypic comparison of drug resistance profiles of clinical tuberculosis isolates using whole genome sequencing in Latvia. BMC Infect Dis. 2023;23(1):638. 37770850 10.1186/s12879-023-08629-7 PMC10540372

[35] Enkirch T, Werngren J, Groenheit R, et al. Systematic review of whole-genome sequencing data to predict phenotypic drug resistance and susceptibility in Swedish *Mycobacterium tuberculosis* isolates, 2016 to 2018. Antimicrob Agents Chemother. 2020;64:e02550–19. 32122893 10.1128/AAC.02550-19 PMC7179598

[36] Zhang M, Lu Y, Zhu Y, et al. Whole-genome Sequencing to Predict *Mycobacterium tuberculosis* Drug Resistance: A Retrospective Observational Study in Eastern China. Antibiotics. 2023;12:1257. 37627677 10.3390/antibiotics12081257 PMC10451829

[37] Guan C, Wang M. Diagnostic yield of nine user-friendly bioinformatics tools for predicting *Mycobacterium tuberculosis* drug resistance: a systematic review and network meta-analysis. PLOS Glob Public Health 2025;5(4). 10.1371/journal.pgph.0004465 PMC12011222 40258039

[38] Wiscovitch-Russo R, Dupont CL. Whole-genome sequencing-based genetic diversity, transmission dynamics, and drug-resistant mutations in *Mycobacterium tuberculosis* isolated from extrapulmonary tuberculosis patients in the West. Front Public Health. 2024;9:12. 10.3389/fpubh.2024.1399731 PMC11341482 39185123

[39] Wu X, Gao R, Shen X, et al. Use of whole-genome sequencing to predict *Mycobacterium tuberculosis drug resistance* in Shanghai, China. Int J Infect Dis. 2020;96:48–53. 32339720 10.1016/j.ijid.2020.04.039

[40] Hiebert M, Sharma MK, Rabb M, et al. Mutations in *embB406* Are Associated with Low-Level Ethambutol Resistance in Canadian *Mycobacterium tuberculosis* Isolates. Antibiotics. 2024;13:7. 10.3390/antibiotics13070624 PMC11273804 39061306

[41] Liu W, Chen J, Shen Y, et al. Phenotypic and genotypic characterization of pyrazinamide resistance among multidrug-resistant Mycobacterium tuberculosis clinical isolates in Hangzhou, China. Clin Microbiol Infect. 2018;24(9):e10161–5. 10.1016/j.cmi.2017.12.012 29288021

[42] Liu L, Jiang F, Chen L et al. The impact of combined mutations in the *inhA* and *ahpC* genes on high levels of isoniazid resistance among *katG* non-315 multidrug-resistant tuberculosis isolates from China. Emerg Microbes Infect 2018;7(1):183. 30446638 10.1038/s41426-018-0184-0 PMC6240042

[43] Mok S, Roycroft E, Flanagan PR, et al. Overcoming the challenges of pyrazinamide susceptibility testing in clinical *Mycobacterium tuberculosis* isolates. Antimicrob Agents Chemother. 2021;65(8):1–8. 10.1128/AAC.02617-20 PMC8284449 33972244

