# Supplementary material for: First-line drug-resistant tuberculosis among children under 15 years in Ethiopia: insights from phenotypic and whole-genome sequencing approaches
Source: BMC Infect Dis. 2026 Mar 31;26:928. doi: 10.1186/s12879-026-13206-9 (PMC13162396; doi:10.1186/s12879-026-13206-9)
Supplement: Supplementary file 1 — Supplementary Material 1 [file 12879_2026_13206_MOESM1_ESM.docx]

**Supplement Table 1: Lineage and sub lineage specific associated drug resistance mutation detected by TB-profiler**

| sample | Lineage | Rifampicin | | Isoniazid | | | Ethambutol | | Pyrazinamide | Streptomycin | | |
| --- | --- | --- | --- | --- | --- | --- | --- | --- | --- | --- | --- | --- |
|  |  | rpoB | rpoC | katG | inhA | ahpc | *embA* | *embB* | pncA | *rrs* | Gid | rpsL |
| CH74 | lineage3 | Ser450Leu | - | Ser315Thr |  |  |  | Asp328Gly |  | n.517C>T |  |  |
|  |  |  |  |  |  |  |  | Asp354Ala |  |  |  |  |
| CH64 | lineage4.2.1 | - |  | Ser315Thr |  |  |  | - | - | - | - | - |
| CH78 | lineage3 | His445Arg, |  | Ser315Thr |  |  |  |  |  |  |  |  |
|  |  | His445Tyr |  |  |  |  |  |  |  |  |  |  |
| CH58 | lineage4.2.2.2 | His445Cys |  | Ser315Thr |  |  | c.-16C>T | Met306Ile |  |  | Gly69Asp | Arg86Pro |
| CH02 | lineage4.2.2.2 | - |  | Ser315Thr |  |  | - | - | - | - | - | - |
| CH69 | lineage4.2.2.2 | Ser450Leu | Asp747Ala | Ser140Asn, | Ser94Ala |  |  | Met306Val | Trp68Gly | n.514A>C |  |  |
|  |  |  |  | Ser315Asn |  |  |  |  |  |  |  |  |
| CH55 | lineage4.2.2.2 | Ser450Leu |  | Ser315Thr |  | - |  | Met306Ile | c.-11A>G |  | Gly69Asp | - |
| CH54 | lineage3 | Ser450Leu |  | Ser315Thr |  | - |  |  |  |  |  | Lys43Arg |
| CH68 | lineage4.2.2.2 | Ser450Leu |  | Ser315Thr |  | - |  | Asp1024Asn, | p.Ala134Va |  | Gly69Asp | p.Lys88Thr |
|  |  |  |  |  |  |  |  | Gly406Ala |  |  |  |  |
| CH53 | lineage4.2.2.2 | Ser450Leu |  | ser315Thr |  |  |  | Gly406Asp,Met306Ile |  |  |  | - |
|  |  |  |  |  |  |  |  |  |  |  |  |  |
| CH57 | lineage3 | Ser450Leu |  | Ser315Thr |  | - |  | Met306Ile | Val139Ala |  |  | p.Lys43Arg |
| CH67 | lineage4.2.2.2 | Ser450Leu | - | p.Ser315Thr |  | - |  | Met306Val | Asp12Ala | n.906A>G | Gly69Asp |  |
| CH77 | lineage2.2.1 | Ser450Leu |  | c.45_46insA |  |  |  | - |  |  | - | p.Lys43Arg |
| CH79 | lineage3.1.1 | Ser450Leu |  | .Ser315Thr |  |  |  |  | c.192_193insA |  |  | p.Lys88Arg |
| CH60 | lineage4.2.2.2 | Ser450Leu |  | Ser315Thr |  | - |  | Met306Ile | - |  | gid p.Gly69 | - |
| CH34 | lineage4.1.2.1 | - |  | Ser315Thr |  |  | - | - | - | - | - | - |
| CH33 | lineage4.2.2.1 | Ser450Leu |  | Ser315Thr |  | - |  |  |  | - | - | p.Lys43Arg |
| CH65 | lineage4.2.2.2 | Ser450Leu |  | Ser315Thr |  | - |  | Asp354Ala | - |  | Gly69Asp | - |
| CH59 | lineage4.6.3 | - |  | - | - | - | - | - | - | - | c.102delG | - |
| CH01 | lineage4.2.2.2 | - |  | Ser315Thr |  |  | - | - | - | - | - | - |
| ET84_S41 | lineage4.2.1 | - |  | c.597dupC |  |  | - | - | - | - | - | - |
| CH48 | lineage4.2.2.2 | Ser450Leu |  | Ser315Thr |  | - |  | Met306Ile | c.-11A>G |  | - |  |
| ET857 | lineage4.2.2.2 | Ser450Leu |  |  |  | c.-81C>T |  |  |  |  | - | - |

**Supplement Table 2: Lineage and sub lineage specific associated drug resistance mutation detected by MTBseq**

| sample | Lineage/sub lineage | Rifampicin | Isoniazid | | Ethambutol | Pyrazinamide | Streptomycin | |
| --- | --- | --- | --- | --- | --- | --- | --- | --- |
|  |  | rpoB | KatG | inhA | *embB* | pncA | *rrs* | rpsL |
| CH74 | lineage3 | Ser450Leu | Ser315Thr |  | Asp328Gly |  | n.517C>T |  |
| CH64 | lineage4.2.1 | - | Ser315Thr |  | - | - | - | - |
| CH78 | lineage3 | His445Arg, | Ser315Thr |  |  |  |  |  |
|  |  | His445Tyr |  |  |  |  |  |  |
| CH58 | lineage4.2.2.2 | His445Arg, | Ser315Thr |  | Met306Ile |  |  | Arg86Pro |
|  |  | His445Tyr |  |  |  |  |  |  |
| CH02 | lineage4.2.2.2 | - | Ser315Thr |  | - | - | - | - |
| CH69 | lineage4.2.2.2 | Ser450Leu | Ser315Asn | Ser94Ala | Met306Val | Trp68Gly | n.514A>C |  |
| CH55 | lineage4.2.2.2 | Ser450Leu | Ser315Thr |  | Met306Ile |  |  | - |
| CH54 | lineage3 | Ser450Leu | Ser315Thr |  |  |  |  | Lys43Arg |
| CH68 | lineage4.2.2.2 | Ser450Leu | Ser315Thr |  | Gly406Ala | Ala134Val | n.906A>G | Lys88Thr |
| CH53 | lineage4.2.2.2 | Ser450Leu | Ser315Thr |  | Gly406Asp,Met306Ile |  |  | - |
|  |  |  |  |  |  |  |  |  |
| CH57 | lineage3 | Ser450Leu | Ser315Thr |  | Met306Ile |  |  | Lys43Arg |
| CH67 | lineage4.2.2.2 | Ser450Leu | Ser315Thr |  | Met306Val | Asp12Ala | n.906A>G |  |
| CH77 | lineage2.2.1 | Ser450Leu |  |  | - |  |  | Lys43Arg |
| CH79 | lineage3.1.1 | Ser450Leu | Ser315Thr |  |  |  |  | Lys88Arg |
| CH60 | lineage4.2.2.2 | Ser450Leu | Ser315Thr |  | Met306Ile |  |  | - |
| CH34 | lineage4.1.2.1 | - | Ser315Thr |  | - | - | - | - |
| CH33 | lineage4.2.2.1 | Ser450Leu | Ser315Thr |  |  |  | - | Lys43Arg |
| CH65 | lineage4.2.2.2 | Ser450Leu | Ser315Thr |  | - | - |  | - |
| CH01 | lineage4.2.2.2 | - | Ser315Thr |  | - | - | - | - |
| CH48 | lineage4.2.2.2 | Ser450Leu | Ser315Thr |  | Met306Ile |  |  |  |
| ET857 | lineage4.2.2.2 | Ser450Leu |  |  |  |  |  | - |
